# Laboratory Calibration of Energy Measurement Systems (EMS) under AC Distorted Waveforms

**DOI:** 10.3390/s20216301

**Published:** 2020-11-05

**Authors:** Daniela Istrate, Abderrahim Khamlichi, Soureche Soccalingame, Jorge Rovira, Dominique Fortune, Martin Sira, Pascual Simon, Fernando Garnacho

**Affiliations:** 1 Electrical Metrology Department, LNE, 78197 Trappes CEDEX, France; Soureche.SOCCALINGAME@lne.fr (S.S.); dominique.fortune6@orange.fr (D.F.); 2 FFII-LCOE, Eric Kandel Street 1, Getafe, 28906 Madrid, Spain; ak@ffii.es (A.K.); jrovira@ffii.es (J.R.); psimon@ffii.es (P.S.); fernando.garnacho@ffii.es (F.G.); 3 Department of Primary Metrology of Electrical Quantities, Czech Metrology Institute, Okružní 772/31, 638 00 Brno, Czech Republic; msira@cmi.cz

**Keywords:** energy measurement, railway system, calibration setup, fictive power source, distorted regime, harmonic waveform, calibration procedure, energy measuring system

## Abstract

Current standard EN 50463-2 indicates the tests and the requirements to be satisfied for an energy measurement system of a traction unit for railway applications. Some of these tests are to be done with several harmonics superposed on the rated voltage, respectively current. However, no calibration systems satisfying the standard requirements were available few years ago. The work performed in the EURAMET project “MyRailS” leads to the development of fictive power sources and reference measurement systems described in this paper. Therefore, it is possible to generate distorted 25 kV-50 Hz voltages with harmonics up to 5 kHz and 90° phase-fired currents up to 500 A with harmonics up to 5 kHz. The generated power is measured by developed traceable reference systems with accuracy better than 0.5%.

## 1. Introduction

The evolving changes in European railway system started with the beginning of the 21st century. In June 2008, the European parliament and council published the Directive n° 2008/57/EC [1] on the interoperability of the rail system within the Community. To establish a single European railway area, the European commission requires that energy billings shall be computed on the actual consumed energy [2,3]. All trains shall be equipped with an energy measurement function (EMF), whose measurement accuracy shall be assessed and periodically re-verified, as required by the standard EN 50463-2 [4].

The first edition of the EN 50463-2 standard came into being in 2007 with several updates since. The necessity to calibrate the voltage, current, and energy measurement functions appeared.

Until recently, energy measurement equipment in the railway sector was checked against the requirements of this standard separately for current, respectively for voltage and metering functions (never together) and at levels much lower than their nominal values. Moreover, these verifications and calibrations were done only in dedicated laboratories, never on board trains due to a missing setup and practice.

It is important to outline that the EN 50463-2 standard was issued based on the AC energy meter for electrical distribution networks. Testing such energy meter with distorted current signal of less than 100 A amplitude and harmonics up to 2 kHz is consistent with the reality. However, these amplitudes and frequencies need to be revised for the railway field. Applying the standard to the railway field characterized by high levels of voltages and currents is not obvious. Calibration setups and procedures, which go beyond the existing procedures developed for pure sinusoidal or continuous regime, are required.

The voltages supplied to trains are often distorted harmonically and subject to ripple. These distortions are much larger than those experienced on the usual electricity network [5]. At the same time, the currents drawn by trains also contain high levels of harmonics, inter-harmonics, ripple, and step changes in magnitude associated with train acceleration and braking. Arcing phenomena, generated by a bad pantograph-to-line contact quality, can further introduce transient distortions and serious over-voltages [6,7,8]. The combination of these effects means that accurate determination of real-time power consumption is needed.

The aim of the performed research is the development of a metrological infrastructure for accurate measurement of energy exchange under railway operating conditions. Calibration setups and a new procedure have been developed in the framework of the European joint research project, MyRailS [9,10,11]. This paper focuses on the reference setups developed for the calibration of energy measurement systems (EMS) working under AC distorted waveforms. The most common AC supply systems have rated voltages of 15 kV at a frequency of 16.7 Hz and 25 kV at 50 Hz.

The developed systems were designed to allow the calibration of the entire energy measurement chain considering, thus, the interactions between voltage and current functions as well as their nominal high values. One of the key element of the developed calibration systems is the fictive power source.

Problems appear when a distorted high current/voltage need to be generated (rich content of high amplitude harmonics) due to technological challenges. There are commercial amplifiers able to generate sinusoidal currents with high amplitudes (tens of amperes) and high frequencies (up to 5 kHz). However, to satisfy the railway requests (up to 25 kV and 500 A) supposes to parallel assembly several current sources, carrying them at high potential and isolating them at 25 kV. This solution has its critical technical, financial and mechanical aspects. Therefore, it is not the adopted approach neither for current, nor for voltage generation.

The developed fictive power sources and presented in this paper rely on the use of magnetic cores that solve the insulation issue.

Generate high distorted currents (hundreds of amperes and few kilohertz) requires magnetic materials that do not saturate and electronic devices accepting the appropriate power. Since the calibration setup for the EMS is supposed to go on-board train, it is necessary to look for compact, transportable equipment. For the current generation part, our approach relies on the use of magnetic materials with linear magnetization curve since it can be controlled in open loop and a direct ratio between the injected and the output current can be established.

An important challenge to generate high voltage 25 kV (50 Hz) and 15 kV (16.7 Hz) with superimposed voltage harmonics is to achieve appropriate voltage transformers with special magnetic cores capable to work for 16.7 Hz fundamental component without saturation and to transmit harmonics components up to 5 kHz.

The paper presents the metrological characterized reference systems for laboratory calibration of the EMS under railway AC supply, which include the calibration of voltage and current transducers, digitizer and the algorithm for power and energy measurements. The sources of the calibration setups are able to generate fictive power in distorted regimes with a frequency content up to 5 kHz with a voltage up to 25 kV and a current up to 500 A (close to the on-site conditions). The reference systems can calibrate commercial EMS designed for 15 kV-16.7 Hz and 25 kV-50 Hz standard AC railway supplies [12,13].

## 2. Calibration Setups

A general setup used to calibrate EMS is composed of:

- power source that should generate high voltages and high currents as required in the current standard EN 50463-2;
- reference measurement system (voltage, respectively current sensors, digitizers and data treatment algorithm to determine the requested quantities);
- device under test.

Two calibrations setups are presented in this paper. LNE calibration setup outlines the generation of high currents with harmonic content up to 5 kHz, while the LCOE calibration setup presents the generation of high voltages and high currents with harmonics in both up to 5 kHz.

### 2.1. LNE Calibration Set-Up

**Figure 1:**
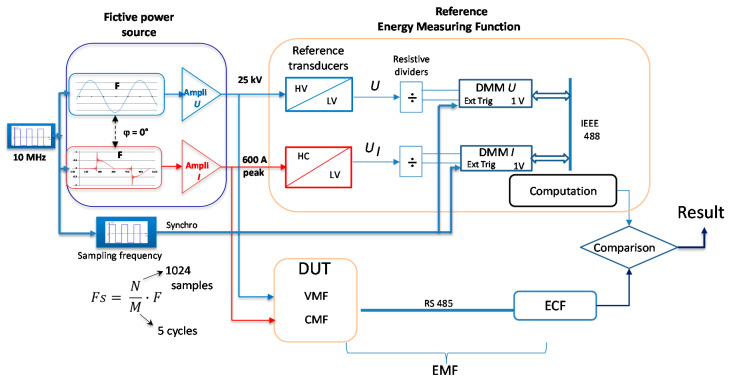
Design principle of energy measurement systems (EMS) calibration setup developed by LNE.

**Figure 2:**
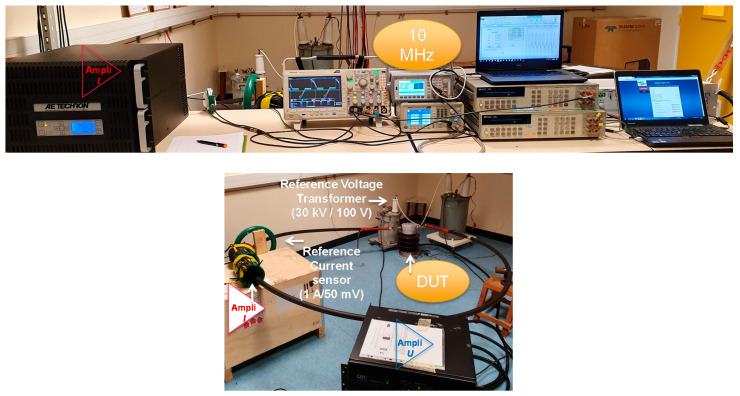
EMS calibration setup at LNE.

The design principle of the EMS calibration setup is illustrated in Figure 1, while a picture of it is given in Figure 2.

The fictive power source generates voltage and current waveforms starting from numerical low-level voltages that are amplified further and, respectively, converted into current.

A 10 MHz signal is used to synchronize voltage and current generation and to trigger the signal acquisition. This phase-locked loop allows the synchronization of the calibration system and, thus, the control of the phase displacement between voltage and current for the generation part.

The high phantom power (less than 10 MW) is composed of the high current (distorted or sinusoidal) flowing through a shielded cable that is mounted at the high potential (sinusoidal). This power applies to the reference system and to the device under test. The calibration results are the corrections of the device under test in terms of energy percentages.

#### 2.1.1. Current Generation with Arbitrary Waveforms

**Figure 3:**
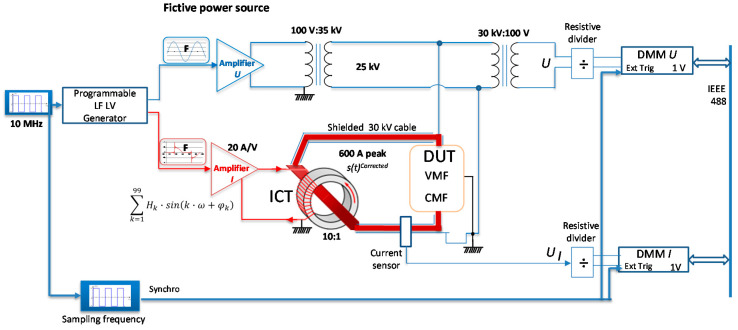
Principle scheme of the LNE current generation and fictive power source.

The distorted current necessary to calibrate an EMS according to the EN 50463-2 standard is generated as illustrated in Figure 3. Low level voltage with numerically created arbitrary waveform is provided by a programmable low frequency (30 MHz) generator. Further, this voltage is amplified and converted into current by means of the Amplifier *I* which is a high-power amplifier featuring a gain of 20 A/V, a robust output (90 A, 45 V for 0.5 Ω load) and a power range over 4000 watts. The amplified current supplies a wideband injection current transformer (ICT) with a ratio of 10:1. The device under test (DUT) will have a current flowing through it 200 times the initially generated voltage.

This setup allows generating high currents with important harmonic content if the operation point of the injection current transformer, ICT, is optimized. This assumes finding a compromise between the transformation ratio, the impedances of the primary and secondary circuits, as well as the power supply mode of the transformer in order to avoid saturation of its magnetic core.

Since the impedance of the secondary loop is transferred to the primary with the square of the ICT ratio, the lower impedance, ratio the better. To reach this, we acted on the cable parameters: Copper instead of Aluminium conductor and 3 m length cable. However, certain limitations apply: the length of the cable should take into account the on-board calibrations, while the secondary loop impedance contains an unknown value, which is the impedance of the DUT.

The high voltage generation part of the LNE system is composed of the low level waveform generator, an audio amplifier (Amplifier U in Figure 3) and the step up potential transformer (100 V: 35 kV). The high potential (ex. 25 kV, 50 Hz) is applied to the cable carrying the high current. A high-voltage shielded cable is used in order to reduce the influence of high voltage on the current measurement. The cable sheath is maintained at zero potential, avoiding any voltage stress to the reference current sensor.

#### 2.1.2. Reference Measuring System

The reference energy measuring function relies on a traceable and synchronized measurement of voltage, respectively current applied to the DUT simultaneously. The reference electrical power is then computed based on a Fast Fourier Transform algorithm.

The voltage reference transducer is a step-down instrument transformer, while the current reference transducer is an inductive type of current monitor with 5 MHz bandwidth and 25.4 mm inner diameter (suitable for cables with a large cross section). Two traceable resistive dividers are used to adapt the level of the voltages for the 1 V range of the digital multimeters (DMMs) in order to take advantage of the high accuracy and stability of this range. All these components are calibrated and traceable to the LNE national standards.

The voltage and current acquisitions are synchronized as illustrated in Figure 1. The sampling frequency is calculated according to the fundamental frequency of the signal to be measured, F, the number of points, N, and periods to be recorded, M. One acquisition gets the data corresponding to 5 cycles. The operator starts the acquisition and stops it after the desired number of repetitions (the limit is given by the performances of the acquisition device and software, 2^20^ repetitions).

#### 2.1.3. Characterization and Corrections Implementation

The setup components might introduce attenuation and phase displacement on the input signal. A proper characterization allows identifying the corrections to be implemented in order to compensate the influence of the setup.

**Figure 4:**
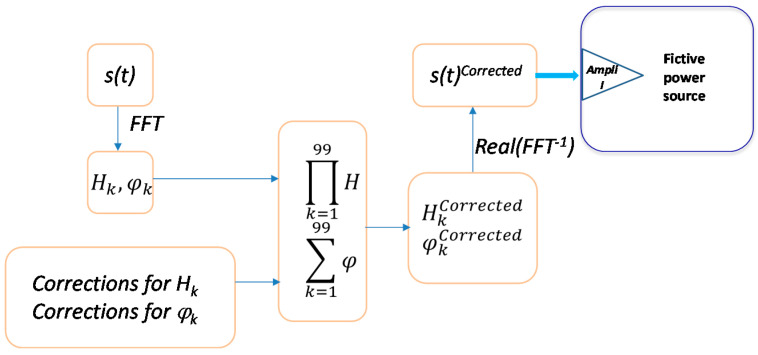
Diagram to apply corrections.

The current generation and measurement part (Figure 3) that was characterized involves the Amplifier I, the ICT, and the Current sensor. A traceable, known and sinusoidal input voltage supplied by a calibrator was injected in the Amplifier I while a calibrated DMM measured the output of a current sensor. The frequency of the input voltage varied between 50 Hz and 5 kHz with a 50 Hz step. The transfer function (gain and phase shift) with the frequency was obtained. The gain of the transfer function is the ratio between the output amplitude of the current sensor and the input voltage amplitude. The current part of the LNE setup attenuates with more than −3 dB frequencies higher than 2 kHz and shift the phase of the input signal with maximum 80° for the whole frequency range. Corrections of the generated waveform are implemented according to these characterization results as illustrated in Figure 4.

Any periodic input signal, *s*(*t*) can be decomposed into a sum of sinusoidal signals by applying the Fourier transform:

(1) $$s\left(t\right)=\;H_{0}+\overset{99}{\underset{k=1}{\sum }}H_{k}sin\left(2\pi k\frac{t}{T}+\varphi_{k}\right)$$

with *H*_0_, *H_k_*—the modules of the continuous component, respectively of the harmonics order *k*, *φ_k_*—the arguments of the harmonics *k* with respect to the fundamental, *T*—the period of the fundamental component.

The correction factors for harmonic modules are obtained as ratio between the fundamental module and the gain obtained during the characterization. Therefore, a unitary correction factor corresponds to the fundamental component and its value increases with the frequency going up to 2.6 for 5 kHz component. The amplitude of each harmonic composing the targeted waveform is multiplied by the appropriate correction factor. The phase of each harmonic is adjusted by extracting the value of the phase shift determined during the characterization from the phase value provided by the FFT of the targeted waveform. Thus, new values for amplitudes, $H_{k}^{Corrected}$ and phases, $\varphi_{k}^{Corrected}$ are obtained. The Inverse Fourier Transform allows getting the discrete corrected waveform to be generated, *s*(*t*)*^Corrected^*.

The developed and characterized setups are planned to be used as often as an EMS calibration is required. The generation part and the reference system including the high voltage and high current cable will not change, at least for laboratory calibrations. This process (downstream measurements inducing upstream corrections) is a form of feedback which is possible due to the slow evolution of the system. Even if currently the evaluation of the corrections to be implemented is carried out once for an operating point, it is finally planned to dynamically evaluate the error term in order to be able to compensate the system in regular time.

### 2.2. FFII-LCOE Calibration Set-Up

#### 2.2.1. Scheme of the Calibration Set-Up

**Figure 5:**
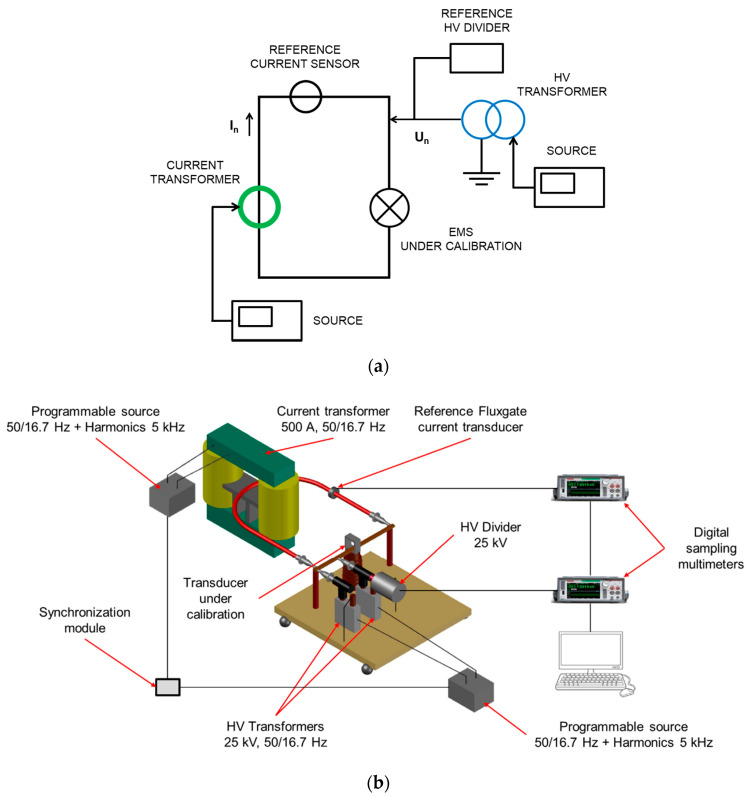
(**a**) Conceptual circuit, (**b**) Scheme of the calibration setup developed by LCOE.

An AC fictive power source has been developed by LCOE for generating voltage and current waveforms (15 kV-16.7 Hz or 25 kV-50 Hz for voltage and 500 A for current) with harmonics up to 5 kHz for voltage or current outputs. Figure 5 shows the conceptual circuit and the layout scheme of the calibration set-up. It consists of a current loop injected through a current transformer and a high voltage circuit injected through high voltage transformers. Two voltage sources are used for feeding voltage or current transformers.

#### 2.2.2. Calibration Setup Implementation

**Figure 6:**
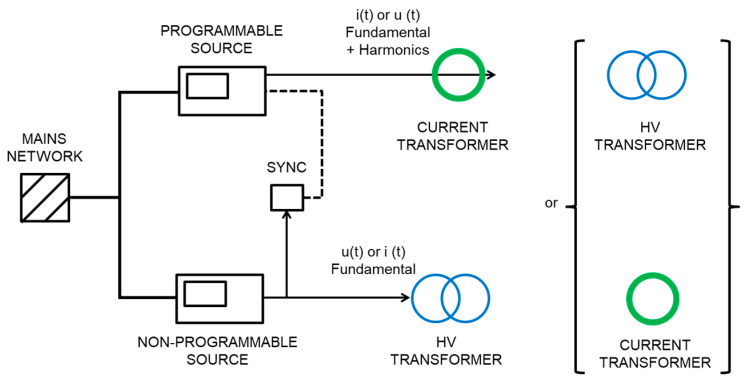
Low voltage circuits used for generating both voltage u(t) and current i(t) waveforms using a programmable for arbitrary waveforms and the other one for the fundamental waveform.

The current loop is energized by means of a commercial current transformer up to 4 kA/160 A (50 Hz) and the high voltage circuit by two identical commercial high voltage measuring transformers (22 kV/110 V) connected in series or in parallel to the bus bar of the current loop. Two low voltage sources (300 V; 16 A) are used: a programmable source works as an arbitrary waveform generator to feed voltage or current transformers, to generate the fundamental component with harmonic components in only one circuit (current or voltage circuit) and other low voltage source is used for the fundamental waveform, as it is shown in Figure 6. When a circuit is using the programmable source, the other circuit is working at the fundamental frequency component by means of a non-programmable source. A synchronization module achieves a stable angle displacement between fundamental voltage and current waveforms. Frequency from the non-programmable source is entered in the input of the module to synchronize the programmable source.

The attenuation and phase displacement caused by the voltage or current transformers when harmonic components are generated are compensated by software in the programmable voltage source modifying the signal to be generated. For this purpose, a previous frequency response analysis of both current and voltage transformers was performed to determine the correction factors to be applied for each harmonic component to be generated. The target phase-fired current signal was achieved, generating a distorted original waveform in the programmable source with over amplitudes for harmonic components on the basis of the referred correction factors to compensate the attenuation and the phase displacement of each harmonic component.

**Figure 7:**
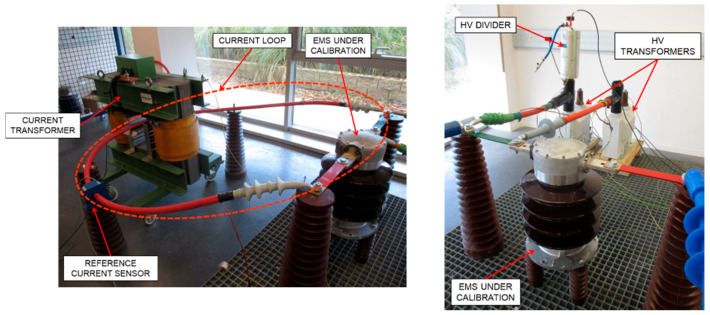
Lay-out of the high current circuit and the high voltage circuit when an EMS is under calibration.

A reference fluxgate current sensor (636 A; 1500/1) with a shunt of 10 Ω and a high voltage reference resistive-capacitive divider (25 kV; 1060/1; 10 kHz, 0.2%) are used to measure both current and voltage quantities. The EMS under calibration is fed by both current and voltage circuits (see Figure 7).

**Figure 8:**
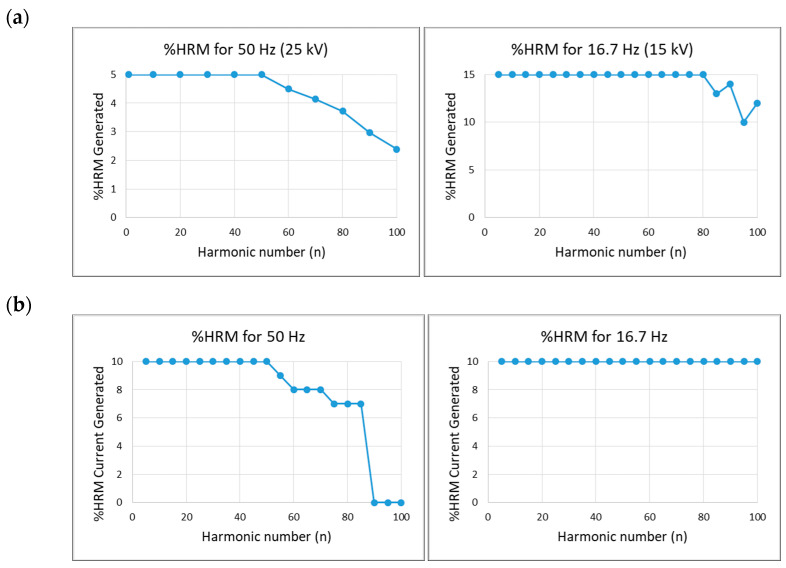
(**a**) Limits for voltage harmonic components, (**b**) Limits for current harmonic components. Note: HRM = Harmonic.

The harmonics limits achieved by current and voltage transformers are shown in Figure 8. The harmonic content that this calibration setup is able to generate for voltage waveforms is 5% up to harmonic number n = 50 (2.5 kHz) for 25 kV–50 Hz and 10% up to n = 100 (5 kHz) for 15 kV–16.7 Hz. The maximum percentage of harmonic content (%HRM) is represented for each harmonic number (n).

Two identical multimeters that operate as digitizers (1MS/s) are used to record both voltage and current signals. The multimeter of voltage signal works as Master to control the trigger of the other multimeter used for the current signal. A specific measuring software was developed by FFII-LCOE to measure active and reactive energy for sinusoidal waveforms and non-active energy for non-sinusoidal waveforms, according to the model functions described in Section 3.

**Figure 9:**
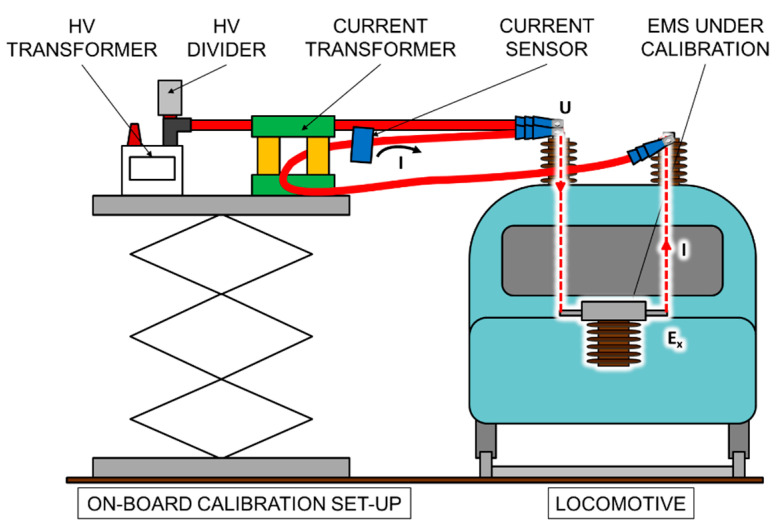

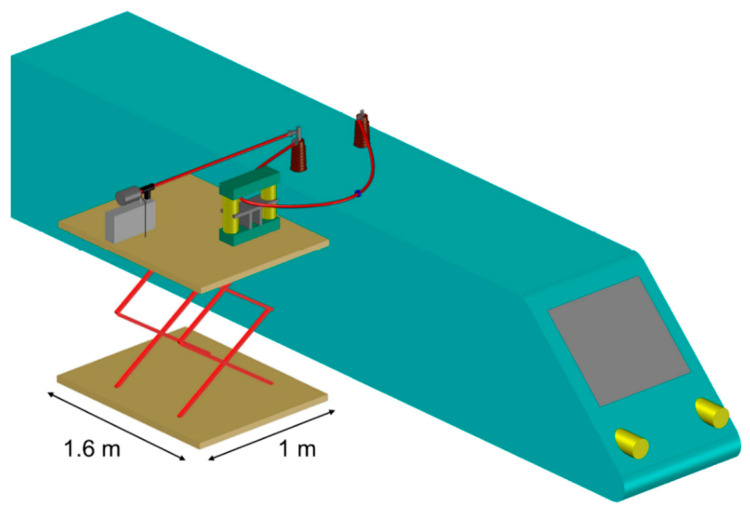
On-board calibration setup.

This setup has been designed also to be disposed in a mobile platform for on-board calibrations, as Figure 9 shows.

## 3. Uncertainty Estimation

The main aim of any calibration is to obtain the corrections (the same value as the errors but with the opposite sign) to be applied to the Device Under Test (DUT) for an appropriate use. The corrections plus their uncertainty should provide measurement results situated in the limits of the maximum permissible errors for an accepted device. Generally, these limits are given by the standards. Particularly, the EN 50463-2 [4] provides the limits for the energy measurement on board trains.

### 3.1. Model Functions for Uncertainty Estimation of Active and Reactive Power and Energy

Formulas for uncertainty estimation of RMS values of voltage and current, active power, apparent power, non-active power, active energy, non-active energy, and reactive energy are summarized below:

(2) $$u\left[V_{RMS}\left(t_{j}\right)\right]=\sqrt{u^{2}\left(\delta_{v1}\right)+{\left[\frac{\left|\bar{V\left(t_{j}\right)}\right|}{V_{RMS}\left(t_{j}\right)}\cdot \frac{U_{FS}}{V_{RMS}\left(t_{j}\right)}\right]}^{2}\cdot u^{2}\left(\delta_{v2}\right)+\underset{j}{\sum }u^{2}\left(\delta_{j,VD}\right)+\underset{j}{\sum }u^{2}\left(\delta_{j,t}\right)}$$

(3) $$u\left[V_{RMS}\left(t_{j}\right)\right]=\sqrt{u^{2}\left(\delta_{v1}\right)+{\left[\frac{\left|\bar{V\left(t_{j}\right)}\right|}{V_{RMS}\left(t_{j}\right)}\cdot \frac{U_{FS}}{V_{RMS}\left(t_{j}\right)}\right]}^{2}\cdot u^{2}\left(\delta_{v2}\right)+\underset{j}{\sum }u^{2}\left(\delta_{j,VD}\right)+\underset{j}{\sum }u^{2}\left(\delta_{j,t}\right)}$$

(4) $$\begin{array}{c} u\left[I_{RMS}\left(t_{j}\right)\right]\\ =\sqrt{u^{2}\left({\delta^{\prime }}_{v1}\right)+{\left[\frac{\left|\bar{I\left(t_{j}\right)}\right|}{I_{RMS}\left(t_{j}\right)}\cdot \frac{I_{FS}}{I_{RMS}\left(t_{j}\right)}\right]}^{2}\cdot u^{2}\left({\delta^{\prime }}_{v2}\right)+\underset{j}{\sum }u^{2}\left(\delta_{j,CT}\right)+\underset{j}{\sum }u^{2}\left(\delta_{j,R_{s}}\right)+\underset{j}{\sum }u^{2}\left({\delta^{\prime }}_{j,t}\right)} \end{array}$$

(5) $$\begin{array}{c} u\left[P\left(t_{j}\right)\right]\\ =\sqrt{\begin{array}{c} \left[u^{2}\left(\delta_{v1}\right)+\underset{j}{\sum }u^{2}\left(\delta_{j,VD}\right)+u^{2}\left({\delta^{\prime }}_{v1}\right)+\underset{j}{\sum }u^{2}\left(\delta_{j,CT}\right)+\underset{j}{\sum }u^{2}\left(\delta_{j,R_{s}}\right)\right]+\underset{j}{\sum }u^{2}\left(\delta_{j,t}\right)\\ +\underset{j}{\sum }u^{2}\left({\delta^{\prime }}_{j,t}\right)+\frac{U_{FS}^{2}\cdot {\left|\bar{I\left(t_{j}\right)}\right|}^{2}}{P^{2}\left(t_{j}\right)}\cdot u^{2}\left(\delta_{v2}\right)+\frac{I_{FS}^{2}\cdot {\left|\bar{V\left(t_{j}\right)}\right|}^{2}}{P^{2}\left(t_{j}\right)}\cdot u^{2}\left({\delta^{\prime }}_{v2}\right) \end{array}} \end{array}$$

(6) $$u\left[S\left(t_{j}\right)\right]=\sqrt{u^{2}\left[V_{RMS}\left(t_{j}\right)\right]+u^{2}\left[I_{RMS}\left(t_{j}\right)\right]}$$

(7) $$u\left[N\left(t_{j}\right)\right]=\sqrt{{\left(\frac{S\left(t_{j}\right)}{N\left(t_{j}\right)}\right)}^{4}\cdot u^{2}\left[S\left(t_{j}\right)\right]+{\left(\frac{S\left(t_{j}\right)}{N\left(t_{j}\right)}\right)}^{4}\cdot u^{2}\left[P\left(t_{j}\right)\right]}$$

(8) $$u\left[E_{P}\right]=\sqrt{u^{2}\left[P\right]+u^{2}\left[T\right]}$$

(9) $$u\left[N_{Q}\right]=\sqrt{u^{2}\left[N\right]+u^{2}\left[T\right]}$$

The notations in these formulas are:

*V_rms_*(*t_j_*), *I_rms_*(*t_j_*), *P*(*t_j_*)—the RMS voltage, current and, respectively active power at *t_j_* instant;
*S*(*t_j_*), *N*(*t_j_*)—the apparent, respectively the non-active power for a period starting at *t_j_* instant;
*E_p_*, *N_Q_*—the active energy, respectively the non-active energy in the measuring interval;
*δ_x_* factors represent the relative errors introduced by the devices composing the setups.

All uncertainty components were identified by calibrations in LNE and FFII-LCOE and experimentally. Thus, for the Voltage Measurement Function (VMF), errors introduced by HV transformers, the HV and resistive dividers, respectively DMM’s errors like the multimeter bandwidth limitation, the converter integration time, the signal quantization, the sampling jitter, and the resolution are all considered.

The uncertainties for the Current Measurement Function (CMF) contain the calibration uncertainty of the conversion coefficient of the current sensor, the influence of the primary conductor position, the cross-talk effect, the current sensor linearity with current level, and with frequency. The DMM uncertainties are considered in a similar way as for the VMF but taken into account the current measurement ranges.

As concerning the active power, the uncertainty related to the phase displacement between voltage and current has to be added. The different sources that can introduce supplementary phase shifts are the voltage instrument transformer, the resistive dividers, the current sensor, and the multimeters. The latter can shift the signals phase because of their bandwidth difference, their integration time difference, their trigger delay and their sampling jitter. In addition to all these sources of error, there is also the phase shift due to the quantization of the signals.

### 3.2. Uncertainty Budget

The estimation of the uncertainty was performed using two methods: (1) GUM uncertainty framework (GUF) according to [14] and (2) Monte Carlo method according to [15].

A calibration in terms of active power will generate the following result:

(10) $$Correction\;\left(W\right)=P_{ref}-P_{DUT}$$

where *P_ref_* is the power indicated by the reference system; *P_DUT_* is the power indicated by the Device Under Test.

The relative value of the combined standard uncertainty associated with *Correction* can be obtain by applying the rules of the Guide to the expression of uncertainty [11]:

(11) $$\frac{u_{c}\left(Correction\right)}{Correction}=\sqrt{\frac{u^{2}\left(P_{DUT}\right)}{P_{DUT}^{2}}+\frac{u^{2}\left(P_{Ref}\right)}{P_{Ref}^{2}}}$$

with *u*(*P_DUT_*)—the standard uncertainty of the power indicated by the DUT. This component is given by the standard deviation of the number of performed repetitions for a given power; *u*(*P_Ref_*)—the standard uncertainty of the power indicated by the reference system. Its estimation is performed according to the description of Section 3.1.

In sinusoidal regime, the active power is defined by:

(12) $$P_{Ref}=U_{Ref}\cdot I_{Ref}\cdot \cos\left(\varphi_{Ref}\right)$$

where *U_Ref_*, *I_Ref_* are the RMS values of the *u*(*t*), respectively *i*(*t*) signals and *φ_Ref_* represent their phase shift. These three quantities are independent.

The relative uncertainty will therefore be expressed as a function of the apparent power *S = UI* in the form:

(13) $${\left(\frac{u\left(P_{Ref}\right)}{S}\right)}^{2}=\left[{\left(\frac{u\left(U_{Ref}\right)}{U_{Ref}}\right)}^{2}+{\left(\frac{u\left(I_{Ref}\right)}{I_{Ref}}\right)}^{2}\right]\cdot cos^{2}\left(\varphi_{Ref}\right)+sin^{2}\varphi_{Ref}\cdot u^{2}(\varphi_{Ref})$$

Considering all the uncertainty components cited in Section 3.1 and different power factors, the relative standard uncertainties illustrated in Table 1 are obtained.

The relative value of the expanded uncertainty (for a coverage probability of 95.45% and a coverage factor k = 2) of active power measurement for reference systems is 0.1% for sinusoidal signals of high amplitudes (25 kV, 400 A).

The developed fictive power sources are able to generate distorted voltage and current signals. The relative values of the expanded uncertainty obtained with GUF method are given in Table 2 for different distorted signals.

The GUF method is simplified and assumes that the uncertainty of output quantity depends linearly on the uncertainties of input quantities. This can cause overestimated results. Contrarily, the Monte Carlo method can propagate probability density functions of input uncertainties and linearity is not required. The result of Monte Carlo calculation is a probability density function and not just a value.

The Monte Carlo method was applied to calculate the uncertainty of *P_Ref_*. The probability density function of all input quantities was considered as normal thus representing the same input uncertainties as used in the GUF method. The calculation itself used the same equations as for the GUF method. The Monte Carlo method works by repeating the calculation of output uncertainties many times with input uncertainties randomized according probability density functions. The number of repetitions (cycles) has to be found out in every particular case. The value of relative uncertainty of *u*(*P_Ref_*)/*S* was stable to 1% for the number of Monte Carlo cycles greater than 10^5^. For all final calculations, the number of Monte Carlo cycles was set to 10^6^.

First a simplified case was considered. Simple sine waveform was used for both voltage and current. The obtained uncertainty of *u*(*P_Ref_*)/*S* calculated by Monte Carlo method follows a normal distribution and is very similar to the value calculated by GUF as outlined in Table 3.

This result validates the uncertainty calculation for the active power in simplified case and can be viewed as a check of correctness for both GUF and Monte Carlo methods.

**Figure 10:**
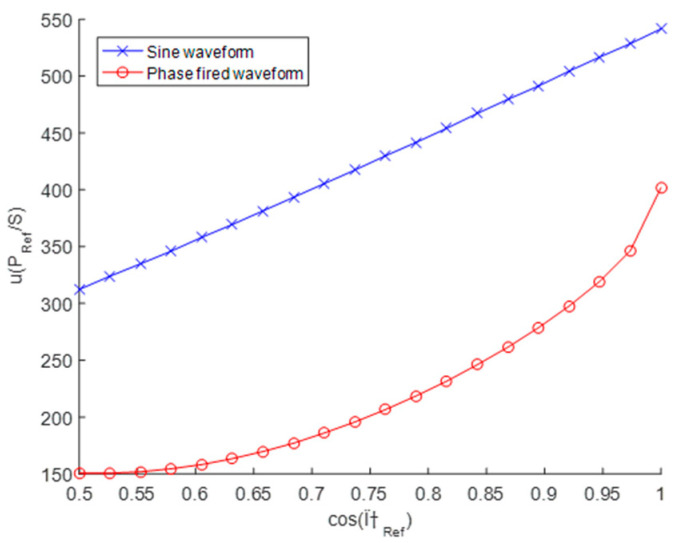
Active power uncertainties relative to the apparent power computed with Monte Carlo method.

For the case of 90° phase-fired waveform, the relative uncertainty calculated by Monte Carlo was significantly lower, 25% to 50% smaller than values of Table 2. Figure 10 shows a comparison of the uncertainty of *u*(*P_Ref_*)*/S* calculated by Monte Carlo for both sine wave and 90° phase-fired current waveform at different values of $cos\left(\varphi_{Ref}\right)$. While the uncertainties calculated using GUF (or Monte Carlo with sine waveform) are linearly dependent on the $cos\left(\varphi_{Ref}\right)$, the dependence of uncertainties calculated for phase fired waveform shows very nonlinear behaviour. The smaller uncertainties can be contributed to the nonlinear character of the equations manifesting in the case of complex waveform. Up to it, the special shape of phase fired waveform causes the output uncertainty is decreasing faster for higher values of $cos\left(\varphi_{Ref}\right)$.

The GUF method presents the advantage of very quick calculations of uncertainty. The uncertainty is overestimated however it is correct and acceptable approach if fast uncertainty estimate is required. Otherwise a Monte Carlo calculation should be performed, unfortunately it requires considerable computer resources and a lot of time. The result of a Monte Carlo calculation is a distribution function, therefore, accurate information and more details about the variation of the studied quantity is obtained. The smaller achieved uncertainties for 90° phase-fired waveform consolidate this statement.

The results for sine waveform (blue line) are very similar the results calculated by GUF. The red line represents results for 90° phase-fired waveform.

## 4. Calibration Procedure

This calibration procedure has been developed in order to be applied to commercial Energy Measuring Systems, which are installed in railway systems. This procedure could be applied for laboratory and also for on-site calibrations.

1. Before carrying out any test, both voltage and current waveforms have to be synchronized to control the phase displacement between both signals.
2. Accuracy test without harmonic content: Voltage and current waveforms at the fundamental frequency of the EMS are applied to the current loop.

Measured EMS errors (ε_EMS_) shall not exceed the limits given in Table 4 due to variations in input quantities (current, voltage and power factor or sinφ) given in this table. U_n_ and I_n_ are the rated primary voltage, respectively current of the EMF. These error limits shall apply to the measurement of energy in each direction (generated or consumed).

**Figure 11:**
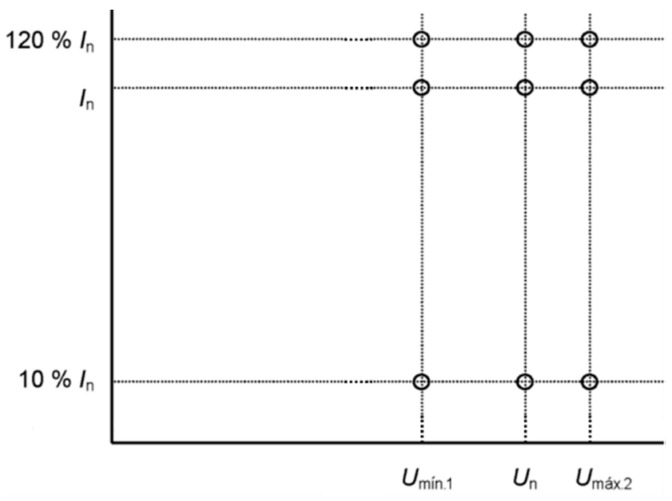
Calibration points for EMS without harmonics.

The calibration points are shown in Figure 11.

U_min2_, U_min1_, and U_max2_ voltage values are given in EN 50163 [13], depending on the rated voltage of the EMS under calibration.

3. Accuracy test with harmonics in the voltage circuit: This test is carried out applying different harmonic contents in the voltage waveform. The ε_EMS_ error with harmonic components in the voltage waveform shall also be measured at the points indicated in Table 5.

Fundamental and harmonic voltages are in phase.

4. Accuracy test with harmonics in the current circuit: this test is carried out applying different harmonic contents in the current waveform. The ε_EMS_ error with harmonic components in the voltage waveform shall also be measured at the points indicated in Table 6.

Fundamental and harmonic currents are in phase.

**Figure 12:**
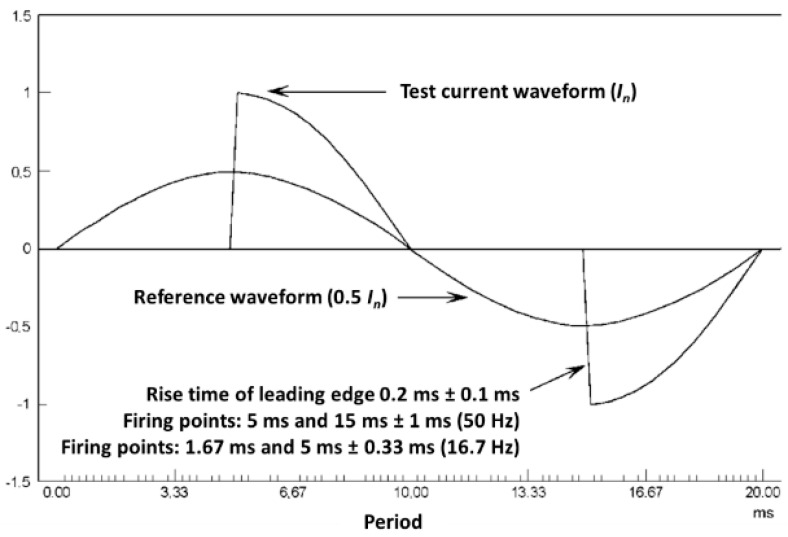
Phase-fired current waveform (90°).

5. Accuracy test of the influence of odd harmonics in the current circuit (phase-fired waveform): The ε_EMS_ error with the phase-fired current waveform shall be also measured at the points indicated in Table 7. The current waveform is shown in Figure 12.

The calibration shall include tests with 45°, 90°, and 135° phase-fired waveforms.

**Figure 13:**
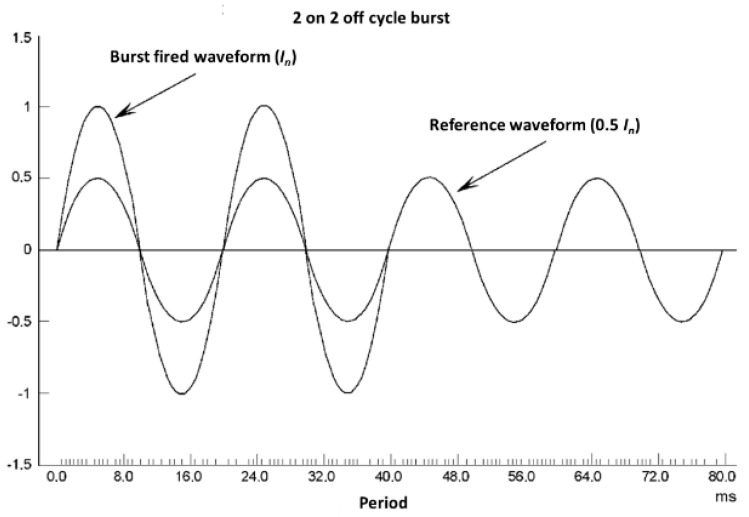
Burst-fired current waveform.

6. Accuracy test of the influence of inter-harmonics in the current circuit (burst-fired waveform): The ε_EMS_ error with the burst-fired current waveform shall be also measured at the points indicated in Table 8. The current waveform is shown in Figure 13.

**Figure 14:**
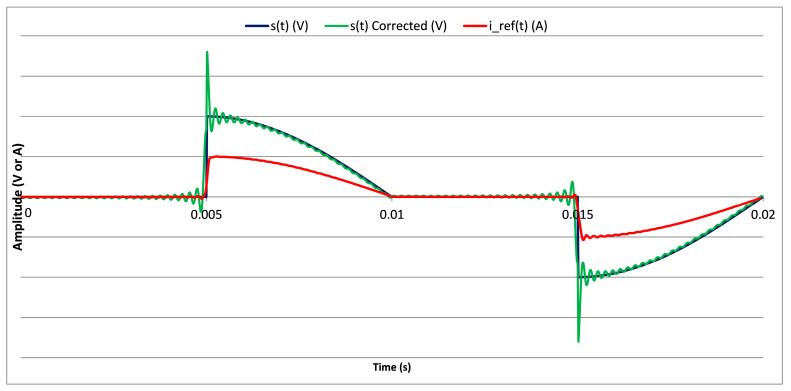
Targeted, corrected and acquired 90° phase-fired waveform.

The results of an EMS calibration using the developed setups and the implemented corrections are illustrated in Figure 14. Targeted, corrected and acquired 90° phase-fired waveform The *s*(*t*) *[V]* curve represents the targeted waveform, the *s*(*t*) *Corrected [V]* curve is the waveform to be generated and obtained once the corrections were implemented. These two curves are obtained with the same amplitude adjusted on the Programmable LF LV generator. The curve *i_ref*(*t*) *[A]* is the image of the current as it is provided by the reference current sensor output. Since the Y-axis scale is not the same, the three curves are plotted without values and mainly to outline the effect of the application of corrections. The peak and oscillations present in the signal to be generated, *s*(*t*) *Corrected* are corrected and the obtained signal that feeds the DUT has the waveform of the targeted signal.

The developed setups are versatile. One can use them to generate the distorted waveforms without or with implementing corrections. There is a trade-off between the maximum reachable amplitude of the generated signal and the high frequency limit for undistorted harmonics. Implementing the corrections allows reaching a 5 kHz non-attenuated frequency but limits the amplitude of the generated signals to 500 A.

## 5. Conclusions

The power and energy measurement accuracy under distorted conditions is an important achievement that the European Commission requires in order to establish a single European railway area. To answer this requirement, two reference calibration set-ups have been developed by LNE and FFII-LCOE for laboratory calibration of EMS installed in locomotives.

The main technical improvements are the two developed setups for the energy measurement systems. Generating 25 kV with harmonics up to 5 kHz, respectively phase-fired current waveforms going up to 500 A and harmonics up to 5 kHz are the most important achievements. The progresses made in the field of material structures allows today to find commercially available compact magnetic material with wideband nanocrystalline cores at more than accessible prices. It is one of the elements which, combined with high power wideband amplifiers, made possible the power generation in a transportable and compact way.

The new calibration facilities developed in this project are designed for on-board calibrations, metrological characterized and have the ability to reproduce waveform distortions answering thus to the EN 50463-2 requirements. A new procedure has been developed for calibration of EMS. This procedure has been applied for calibration of commercial EMS.

These developments will contribute to the improvement of the National Metrology Institutes metrological capabilities for the calibration of AC voltage and current transducers and power and energy meters used under harsh electrical conditions. Future work will focus on the implementation of a closed loop control to compensate the influence of the setup and of the environmental factors like temperature or electromagnetic interferences.

## Figures and Tables

**Table 1:** Relative standard uncertainties for 25 kV, 50 Hz and 400 A, 50 Hz sinusoidal signals.

$cos\left(\varphi_{Ref}\right)$	Uncertainty Sources	Standard Uncertainty	Sensitivity Coefficient	Contribution to the *u*(*P_ref_*)	$\frac{u\left(P_{Ref}\right)}{S}$ (k = 1)
1	$\frac{u\left(U_{Ref}\right)}{U_{Ref}}$	52.7 µV/V	1	52.7	539.2 µW/VA
	$\frac{u\left(I_{Ref}\right)}{I_{Ref}}$	536.6 µA/A	1	536.6	
	$u\left(\varphi_{Ref}\right)$	175.2 µrad	0	0	
0.8	$\frac{u\left(U_{Ref}\right)}{U_{Ref}}$	52.7 µV/V	0.8	42.2	444.0 µW/VA
	$\frac{u\left(I_{Ref}\right)}{I_{Ref}}$	536.6 µA/A	0.8	429.3	
	$u\left(\varphi_{Ref}\right)$	175.2 µrad	0.6	105.1	
0.5	$\frac{u\left(U_{Ref}\right)}{U_{Ref}}$	52.7 µV/V	0.5	26.35	309.4 µW/VA
	$\frac{u\left(I_{Ref}\right)}{I_{Ref}}$	536.6 µA/A	0.5	268.3	
	$u\left(\varphi_{Ref}\right)$	175.2 µrad	0.87	152.4	

**Table 2:** Summary of uncertainties for distorted signals.

Waveform	Expanded Uncertainty
90° phase-fired waveform with phase shift of 0° with voltage	0.23%
45° phase-fired waveform with phase shift of 0° with voltage	0.20%
135° phase-fired waveform with phase shift of 0° with voltage	0.47%
Burst-fired waveform with phase shift of 0° with voltage	0.20%

**Table 3:** Mean values of *u*(*P_Ref_*)*/S* for one standard uncertainty (equivalent to a coverage factor of 1).

$cos\left(\varphi_{Ref}\right)$	1	0.8	0.5
Monte Carlo	541.32	447.38	311.96
GUF	539.2	444.0	309.4

**Table 4:** EMS error limits according to EN 50463-2 [4].

Current Range	Voltage Range	Power Factor Sin φ	Error Limit, Active Energy	Error Limit, Reactive Energy
10% I_n_ ≤ I < 120% I_n_	U_min1_ ≤ U < U_max2_	PF ≥ 0.85 sinφ = 1	1.5%	3.0%

**Table 5:** Influence of the harmonic components in the voltage circuit on the EMS.

Value of Voltage	Value of Current	Phase Shift	Additional Percentage Error	
			For Active Energy	For Reactive Energy
U_0_ = U_n_ with U_5_ = 10% U_n_ and U_5_ = 10% U_n_ + [U_11_ = 3% U_n_^(1)^] or [U_3_=3% U_n_^(2)^]	I_0_ = 50% I_n_ without harmonic	Cos φ = 1 for active; sen φ = 1 for reactive	According to the manufacturer specification (not defined in the standard for EMS)	

Note: Maximum values obtained from real measurements at 50 Hz^(1)^ and 16.7 Hz^(2)^ in railway systems.

**Table 6:** Influence of the harmonic components in the current circuit on the EMS.

Value of Voltage	Value of Current	Phase Shift	Additional Percentage Error	
			For Active Energy	For Reactive Energy
U_0_ = U_n_ without harmonic	I_0_ = 50% I_n_ with I_5_ = 40%I_n_ and I_0_ = 50% I_n_ with I_13_ = 5%I_n_ + [I_11_ = 13%I_n_^(1)^] or [I_4_ = 9%I_n_^(2)^]	Cos φ = 1 for active; sin φ = 1 for reactive	According to the manufacturer specification (not defined in the standard for EMS)	

Note: Maximum values obtained from real measurements at 50 Hz^(1)^ and 16.7 Hz^(2)^ in railway systems.

**Table 7:** Influence of the harmonic components in the current circuit on the EMS.

Value of Voltage	Value of Current	Phase Shift	Additional Percentage Error	
			For Active Energy	For Reactive Energy
U_0_ = U_n_ without harmonic	I_0_ = 50% I_n_ Phase-fired waveform	Cos φ = 1 for active; sin φ = 1 for reactive	According to the manufacturer specification (not defined in the standard for EMS)	

**Table 8:** Influence of the harmonic components in the current circuit on the EMS.

Value of Voltage	Value of Current	Phase Shift	Additional Percentage Error	
			For Active Energy	For Reactive Energy
U_0_ = U_n_ without harmonic	I_0_ = 50% I_n_ Burst-fired waveform	Cos φ = 1 for active; sen φ = 1 for reactive	According to the manufacturer specification (not defined in the standard for EMS)	
